# Psychometric Properties of the BIS/BAS Scales and the SPSRQ in Flemish Adolescents

**DOI:** 10.5334/pb.298

**Published:** 2016-12-20

**Authors:** Laura Vandeweghe, Annelies Matton, Wim Beyers, Myriam Vervaet, Caroline Braet, Lien Goossens

**Affiliations:** 1 Department of Developmental, Personality and Social Psychology, Ghent University, Henri Dunantlaan 2, 9000 Ghent, Belgium; 2 Department of Psychiatry and Medical Psychology, Center for Eating Disorders, University Hospital Ghent, Belgium

**Keywords:** reinforcement sensitivity theory, adolescence, assessment, factor analysis

## Abstract

**Objective:**

Gray’s Reinforcement Sensitivity Theory (RST) is a frequently used model of personality that is relevant to the period of adolescence. However, the psychometric properties of the most frequently used questionnaires to measure the RST-constructs, namely the Behavioural Inhibition System and Behavioural Activation System Scales (BIS/BAS Scales) and the Sensitivity to Punishment and Sensitivity to Reward Questionnaire (SPSRQ), are rarely examined in samples of adolescents. Therefore, the goal of the present study was to examine the two-factor structure, reliability and convergent validity of the BIS/BAS Scales and SPSRQ in a Flemish adolescent community sample.

**Method:**

A sample of 579 adolescents (39.5% boys; 14–19 years) was recruited. The proposed two-factor structure was assessed using Exploratory Structural Equation Modeling. Reliability was evaluated using internal consistency and construct validity was examined with the correlations between the two questionnaires and with the Temperament and Character Inventory–short form (TCI-SF).

**Results:**

After the removal of problematic items, and the addition of correlated errors, all indices indicated a good fit for the two-factor structure of the modified BIS/BAS Scales. For the modified SPSRQ, three fit indices indicated a good model fit, while a fourth fit index was slightly below the cut-off score of an adequate model fit. Internal consistency of both questionnaires was sufficient. In general, the associations with scales of the TCI-SF were as expected, with positive correlations between BIS-related scales, and between BAS-related scales of all three instruments.

**Discussion:**

In Flemish adolescents, the use of a two-factor model to analyze data gathered with the modified BIS/BAS Scales or modified SPSRQ seems appropriate.

## Introduction

The Reinforcement Sensitivity Theory (RST; Gray, 1970, 1987; Gray & McNaughton, 2000) is a frequently used theoretical framework for explaining motivated behaviour and emotion (Smillie, Pickering, & Jackson, 2006) that is very relevant to the period of adolescence (Galván, 2013; Romer & Hennessy, 2007). However, few questionnaires measuring the RST-constructs have been validated in samples of adolescents.

Originally, the RST discriminates between three independent biological systems: the Behavioural Inhibition System (BIS), activated by conditioned aversive signals and leading to inhibition, the Behavioural Activation System (BAS), activated by conditioned appealing stimuli and leading to approach behaviour, and the Fight-Flight System, activated by unconditioned aversive stimuli and leading to defensive aggression or escape behaviour (Gray, 1970, 1987). Most studies focus on the BIS and the BAS, with interindividual differences in the sensitivity of these systems which are thought to reflect the personality dimensions anxiety and impulsivity respectively (Gray, 1987). Individuals with a highly reactive BIS system are expected to experience high levels of anxiety and to be more cautious, while individuals with a highly reactive BAS system are assumed to display high levels of impulsivity and more risk-taking behaviors (Carver & White, 1994).

In 2000, the RST was revised (Gray & McNaughton, 2000). In this revision, the distinction between conditioned and unconditioned stimuli disappeared. As such, the BIS is now assumed to inhibit all ongoing behaviour whenever conflicts arise due to competing motivational objectives, implying that it no longer functions as a punishment system, but rather serves as a system for conflict detection and resolution (Smillie et al., 2006). The BAS is assumed to mediate responses to all appetitive stimuli (Gray & McNaughton, 2000) and the Fight-Flight System, now called the Fight-Flight-Freeze-System, is thought to be responsible for all reactions to aversive stimuli, leading to aggressive behaviour or fear (Gray & McNaughton, 2000).

Instruments measuring the revised RST-concepts are rare and have seldom been validated (Cogswell, Alloy, van Dulmen, & Fresco, 2006). In contrast, several questionnaires have been constructed to measure BIS and BAS sensitivity as defined by the original RST, such as the BIS/BAS Scales (Carver & White, 1994) and the Sensitivity to Punishment and Sensitivity to Reward Questionnaire (SPSRQ; Torrubia, Avila, Molto, & Caseras, 2001). The BIS/BAS Scales consist of one BIS-scale and three BAS-subscales, namely BAS-Drive (the persistent pursuit of desired goals), BAS-Fun Seeking (the desire for new rewards and a willingness to approach potentially rewarding events) and BAS-Reward Responsiveness (the positive responsivity to reward) (Carver & White, 1994). The SPSRQ (Torrubia et al., 2001) discriminates between the facet scales Sensitivity to Punishment (SP) and Sensitivity to Reward (SR), assessing BIS and BAS functioning respectively. This questionnaire differs from the BIS/BAS Scales in the sense that the items of the BIS/BAS Scales measure reactions to general cues of punishment and reward, whereas the items of the SPSRQ are related to specific cues of punishment and reward, which is more in line with the theoretical propositions of the RST (Matthews & Gilliland, 1999; Torrubia et al., 2001).

Several suggestions have been made regarding how to interpret the scores obtained on these questionnaires within the revised RST. According to the revised RST, BIS as conceptualized by the original theory is a combination of both the revised BIS- and Fight-Flight-Freeze-System- sensitivity (Corr, 2004; Harrison, O’Brien, Lopez, & Treasure, 2010). In line with the theory, it has been proposed that the BIS-scale of the BIS/BAS Scales and the SP-scale of the SPSRQ might include items measuring the sensitivity of the revised BIS as well as the Fight-Flight-Freeze-System (e.g., Smillie et al., 2006). Therefore, some authors propose a subdivision of the BIS-scale of the BIS/BAS Scales into a BIS- and a Fight-Flight-Freeze-System- subscale (e.g., Heym, Ferguson, & Lawrence, 2008; Vervoort et al., 2010). An additional complexity of the interpretation within the revised RST relates to the theorized relation between BIS and BAS. According to the original RST, BIS and BAS are two orthogonal systems (i.e., separable subsystems hypothesis) (Pickering, 1997). Therefore, the scales of both the BIS/BAS Scales and the SPSRQ are developed to be uncorrelated (Carver & White, 1994; Torrubia et al., 2001). Due to limited evidence for BIS and BAS as independent systems, the revised RST posited that although the biological substrates of BIS and BAS may be independent, the behavioural outcome will in most cases be determined by a combination of both BIS and BAS (Corr, 2001, 2004; Vervoort et al., 2010).

Although the validity of these questionnaires, originally developed for adult populations, is well-established in adults (Jorm et al., 1998; Torrubia et al., 2001) and in children (Luman, van Meel, Oosterlaan, & Geurts, 2012; Muris, Meesters, de Kanter, & Timmerman, 2005), they have rarely been psychometrically evaluated among adolescents. This is surprising given the frequent use of the BIS/BAS Scales (e.g., Jones, Tai, Evershed, Knowles, & Bentall, 2006; Knyazev, Slobodskaya, & Wilson, 2004; Ko et al., 2008; Loxton & Dawe, 2001) and the SPSRQ (e.g., Delgado-Rico, Río-Valle, González-Jiménez, Campoy, & Verdejo-García, 2012; Matton, Goossens, Vervaet, & Braet, 2015; Moreno-López, Soriano-Mas, Delgado-Rico, Rio-Valle, & Verdejo-García, 2012; Verdejo-García et al., 2010) in this age group, and the importance to measure the constructs among adolescents. Indeed, the role of BIS and BAS might be very important during adolescence given that reward processing peaks during this developmental age period whereas inhibitory capacities are generally lower compared to other age groups (Galván, 2013; Romer & Hennessy, 2007). Furthermore, variations in BIS and BAS sensitivity can explain individual differences in personality and psychopathology (e.g., Harrison, Treasure, & Smillie, 2011; Heym, Kantini, Checkley, & Cassaday, 2015; Soler et al., 2014). Two studies report findings concerning the factor structure of the BIS/BAS Scales in adolescent samples (Cooper, Gomez, & Aucote, 2007; Yu, Branje, Keijsers, & Meeus, 2011) with Cooper et al. (2007) finding evidence for a four-factor model in an Australian population, whereas Yu et al. (2011) found evidence for a two-factor model in a Dutch population. Regarding the SPSRQ there is even less clarity, since, to our knowledge, this questionnaire has never been validated among adolescents until now.

The first goal of the present study was to perform Exploratory Structural Equation Modeling (ESEM) on both scales to examine the two-factor structure in an adolescent sample. Secondly, we investigated the internal consistency of both questionnaires. Thirdly, the convergent validity was examined by looking at the correlations of both questionnaires with each other and with a related measure of temperament. For this purpose, we used the Temperament and Character Inventory – short form (TCI-SF; Cloninger, Svrakic, & Przybeck, 1993; Duijsens & Spinhoven, 2001). The TCI-SF is based on the personality model of Cloninger, which is related to the RST but focuses on the level of personality rather than on the level of biological substrates (Cloninger, 1987; Cloninger et al., 1993). Four temperament factors are discriminated within this model: Harm Avoidance (i.e., inhibition of responses in the face of aversive stimuli), Novelty Seeking (i.e., responding actively to novel stimuli), Reward Dependence (i.e., responding positively to conditioned signals of reward) and Persistence (i.e., hard-working, stable and persistent despite frustration and fatigue) (Cloninger, 1987; Cloninger et al., 1993). Harm Avoidance and Novelty Seeking are seen as the two main dimensions within this model and are theoretically related to BIS and BAS as defined by the original RST (e.g., Carver & White, 1994; Torrubia et al., 2001).

Based on the theoretical assumptions of the RST (Gray, 1970, 1987; Gray & McNaughton, 2000), and previous findings in Dutch adolescents (Yu et al., 2011), we expected that a two-factor model of both the BIS/BAS Scales and the SPSRQ would show an acceptable fit and good internal consistency. As the factor structure of these scales has never been investigated in a sample of Flemish adolescents, we followed the principle of parsimony, which suggests that simpler models should be tested before advancing to more complicated models (American Psychological Association, 1985). It was further hypothesized that within these two-factor models the BIS-scale would be positively related to the SP-scale, while the BAS-scale would be positively related to the SR-scale. In line with the separable subsystem hypothesis, we expect no correlation between scales measuring BIS sensitivity (i.e., BIS-scale and SP-scale) and BAS sensitivity (i.e., BAS-scale and SR-scale). We further hypothesize that measures of BIS sensitivity would be positively related to Harm Avoidance and measures of BAS sensitivity to Novelty Seeking and Reward Dependence, given the theoretical overlap between these constructs. Based on previous research, a positive association was expected between measures of BAS sensitivity and the Persistence-scale (Mardaga & Hansenne, 2007).

## Materials and Methods

### Participants and Procedure

The present study was part of a larger study on temperament (see Matton, Goossens, Braet, & Vervaet, 2013) for which nine Flemish secondary schools agreed to participate. This resulted in a sample of 567 participants (39.5% boys; 14–19 years, *M*_age_ = 15.73, *SD* = 1.37) after the removal of 12 outlier scores (*z*-scores > ± 3). Different educational levels were represented in the sample (i.e., 64.9% general education; 31.9% technical education; 3.0% vocational education; 0.2% missing). All school principals received information about the goal and design of the study and completed active informed consents. Parents were informed about the study via a passive informed non-consent document, meaning that they had to sign the document if they did not want their adolescent to participate. Participants completed active informed consents after receiving general information about the study. The completion of the questionnaires took place during school hours in the presence of a researcher and a teacher. The total study took about one hour in each class. This procedure was approved by the university’s ethical committee.

### Measures

**BIS/BAS Scales.** The Dutch version of the BIS/BAS Scales (see http://www.ekgp.ugent.be/pages/nl/vragenlijsten/BISBAS_scales.pdf) is developed by Franken, Muris, and Rassin (2005) and based on the original self-report scales for adults (Carver & White, 1994). The BIS/BAS Scales were developed to assess dispositional BIS- and BAS-sensitivities and contain 24 items on a four point scale, ranging from ‘totally disagree’ (score 1) to ‘totally agree’ (score 4). Two main scales are distinguished, a BIS-scale (7 items) and a BAS-scale (13 items). The remaining 4 items are fillers.

**Sensitivity to Punishment and Sensitivity to Reward Questionnaire.** The SPSRQ (Torrubia et al., 2001) was developed to assess BIS- and BAS- functioning by the SP- and SR-scales respectively. This questionnaire was translated and adapted for Dutch children and adolescents by Verbeken (2009) and contains 44 items (i.e., 22 items for each scale), answered on a five point scale ranging from ‘never’ (score 1) to ‘always’ (score 5) (see Appendix A). Four items referring to the rewards of drugs, jobs or paying in a restaurant were removed from the original 48-item item questionnaire because these situations were less appropriate for this age group. Secondly, to decrease the difficulty of the items, they were transformed from questions into statements.

**Temperament and Character Inventory – short form.** The TCI-SF (Cloninger et al., 1993; Duijsens & Spinhoven, 2001) consists of 105 items and is developed to measure the previously described temperament dimensions of Novelty Seeking, Harm Avoidance, Reward Dependence and Persistence, as well as three character dimensions (i.e., Self-Directedness; Cooperativeness, and Self-Transcendence). Each of the seven scales consists of 15 items. As Gray’s concepts of BIS and BAS-sensitivity refer to stable traits, only the four temperament scales were included. Each item is formulated as a statement and is answered by marking ‘correct’ (score 1) or ‘incorrect’ (score 0). Internal consistency in the present sample was good for Harm Avoidance (Cronbach’s α = .84), and acceptable for Reward Dependence (Cronbach’s α = .75), Persistence (Cronbach’s α = .71) and Novelty Seeking (Cronbach’s α = .70).

### Data Analytic Plan

The hypothesized two-factor structure of the BIS/BAS Scales and the SPSRQ was tested in M*plus* 7.4 (Muthén & Muthén, 1998–2015) using Exploratory Structural Equation Modeling (ESEM; Asparouhov & Muthén, 2009). ESEM was applied since it integrates the advantages of both a standard confirmatory factor analysis (i.e., assessing the fit of an a priori model using different SEM parameters and specification of correlated residuals) and exploratory factor analysis (i.e., estimations of cross loadings; Marsh, Morin, Parker, & Kaur, 2014; Wiesner & Schanding, 2013). The latter is important since instruments measuring psychological concepts such as BIS and BAS may have cross loadings (Asparouhov & Muthén, 2009; Marsh et al., 2009). Indeed, limited evidence was found for BIS and BAS as completely orthogonal systems. In order to estimate model parameters, we used Robust Maximum Likelihood Estimation on all data. This full-information estimator provides test statistics and standard errors that are robust to non-normality, non-independence of observations and ordered-categorical variables (Muthén & Muthén, 1998–2015), and handles missing values (Graham, 2009), which were missing completely at random as shown by the Missing Completely At Random test (Little, 1988) (BIS/BAS Scales: χ^2^ (454) = 472.21, *p* = .26; SPSRQ: χ^2^ (1765) = 1756.68, *p* = .55).

Model fit was assessed by relative or normed Chi square (chi-square/degrees of freedom; χ²/df), the Comparable Fit Index (CFI), Root Mean Squared Error of Approximation (RMSEA), and Standardized Root Mean square Residual (SRMR) for fit relative to a null-model. Values of a χ²/df ≤ 5 indicate an acceptable model fit, values ≤ 3 point to a good model fit and values ≤ 2 suggest a very good model fit (Kline, 2015; Schumacker & Lomax, 2004). Cut-off values ≥ .90 for the CFI indicate adequate model fit (Hu & Bentler, 1999). Values of the RMSEA ≤ .05 indicate a good model fit, values ≤ .08 point to an approximate model fit and values ≤ .10 suggest an acceptable model fit (Chen, Curran, Bollen, Kirby, & Paxton, 2008). Cut-off values ≤ .08 for the SRMR suggests a good model fit (Hu & Bentler, 1999). Although these cut-off values are frequently used, they should be handled with caution in social sciences, as factor loadings are usually lower in that research area (Heene, Hilbert, Draxler, Ziegler, & Bühner, 2011). In order to improve model fit, modification indices of residual correlations with values greater than .20 were examined. The models were retested after adding correlated residuals and the removal of poorly loading items, defined as having a factor loading below .30 on the expected factor and a factor loading of .30 or more on the opposite factor, or having poor factor loadings on both factors (Cogswell et al., 2006).

Reliabilities of the original scales and modified scales (i.e., removal of poorly loading items) were tested via Cronbach’s alpha. To test the convergent validity of the BIS/BAS Scales and the SPSRQ, Pearson correlations were calculated between both modified questionnaires as well as with the TCI-SF.

## Results

### Internal Structure

**BIS/BAS Scales.** The fit indices of the ESEM models can be found in Table 1. The full 20-item model indicates a good model fit according to SRMR and normed Chi square, an approximate model fit according to RMSEA, but does not show adequate fit according to CFI. Examination of the item pairs indicated high residual correlations between some of the BAS items, more specifically item 3 with item 9 (i.e., very similar wording), item 4 with item 5 (i.e., neighbouring items in the questionnaire and both point to the recurrent striving for a desired goal), item 10 with item 15 (i.e., both about fun here and now), item 9 with item 20 (i.e., both about the pursuit of feeling good) and item 2 with item 22 (i.e., both point to no fear). After removal of poorly loading items (i.e., item 4, item 5, item 10, item 18, item 22, and item 23) and adding two correlated errors within the BAS item pool, a modified 14-item model was constructed. According to the four fit indices, the modified 14-item model points to a good model fit. All items have a high loading (> .30) on one of the factors, and the pattern confirms the expected differentiation of BIS and BAS (see Table 2).

**Table 1:** Fit indices of the two-factor Exploratory Structural Equation Models for the BIS/BAS Scales and the SPSRQ.

	χ²	df	χ²/df	CFI	RMSEA	SRMR

**BIS/BAS Scales**						
Full model (20 items)	439.67***	151	2.91	.82	.06	.05
Modified model (14 items)	135.98***	62	2.19	.94	.05	.04
**SPSRQ**						
Full model (44 items)	1920.80***	859	2.24	.78	.05	.05
Modified model (36 items)	1033.81***	552	1.87	.89	.04	.04

*Note.* Full model = model with all items included, Modified model = model without the poorly loading items and adding correlated residuals; ***p < .001 BIS/BAS Scales, Behavioural Inhibition System and Behavioural Activation System Scales; SPSRQ, Sensitivity to Punishment and Sensitivity to Reward Questionnaire; χ², chi-square; df: degrees of freedom; χ²/df, relative or normed chi square; CFI, Comparable Fit Index; RMSEA, Root Mean Squared Error of Approximation; SRMR, Standardized Root Mean square Residual.

**Table 2:** Exploratory Structural Equation Model of a Two-Factor Solution for the Modified BIS/BAS Scales (reduced to 14 items).

BIS/BAS items	F1 BIS	F2 BAS

2. Even if something bad is about to happen to me, I rarely experience fear or nervousness.	–.39***	.14**
8. Criticism or scolding hurts me quite a bit.	.66***	–.09
13. I feel pretty worried or upset when I think or know somebody is angry at me.	.67***	–.01
16. If I think something unpleasant is going to happen I usually get pretty “worked up”.	.57***	.03
19. I feel worried when I think I have done poorly at something.	.57***	.06
24. I worry about making mistakes.	.55***	.04
3. I go out of my way to get things I want.	–.11*	.48***
7. When I get something I want, I feel excited and energized.	.17***	.42***
9. When I want something, I usually go all-out to get it.	.02	.67***
12. If I see a chance to get something I want, I move on it right away.	–.01	.43***
14. When I see an opportunity for something I like, I get excited right away.	.12*	.42***
15. I often act on the spur of the moment.	.06	.40***
20. I crave excitement and new sensations.	–.14*	.53***
21. When I go after something I use a “no holds barred” approach.	–.08	.50***

*Note.* Loading on expected factor is printed in bold. The two-factor solution of the modified BIS/BAS Scales included 14 items after deleting item 4, 5, 10, 18, 22, and 23 from the initial 20 items. Additionally, 2 correlated errors were added within the BAS item pool. BIS/BAS Scales, Behavioural Inhibition System and Behavioural Activation System Scales *p < .05; **p < .01; ***p < .001.

**SPSRQ.** The fit indices of the ESEM models can be found in Table 1. The full 44-item model indicates a good model fit according to RMSEA, SRMR and normed Chi square, but does not show adequate fit according to CFI. Examination of the item pairs indicated high residual correlations between some of the SR items, more specifically item 17 with item 29 (i.e., both about quick gains), item 21 with item 35 (i.e., both about competition), item 3 with item 5 (i.e., image, center of attention), item 17 with item 1 (i.e., rewards and gains), item 23 with item 25 (i.e., both about pleasant events), item 34 with item 18 (i.e., shy, insecure), item 19 with item 2 (i.e., illegal, forbidden), item 43 with item 27 (i.e., activity), and item 32 with item 22 (i.e., speaking in public). After removal of poorly loading items (i.e., item 4, item 7, item 13, item 14, item 23, item 25, item 27 and item 31) and adding 7 correlated errors, a modified 36-item model was constructed. The modified 36-item model indicates a good model fit according to RMSEA and SRMR, and a very good model fit according to normed Chi square. Although the model improved according to the CFI, it is still slightly below the cut-off of CFI for an adequate model fit. All items have a high loading (> .30) on one of the factors, and the pattern confirms the expected differentiation of SP and SR (see Table 3).

**Table 3:** Exploratory Structural Equation Model of a Two-Factor Solution for the Modified SPSRQ (reduced to 36 items).

SPSRQ-items	F1 SP	F2 SR

1. The good prospect of obtaining a reward motivates me strongly to do some things.	.02	.39***
3. I like being the centre of attention.	–.17**	.51***
5. I spend a lot of time on obtaining a good image.	.07	.49***
9. In a group, I try to make my opinions the most intelligent or the funniest.	.08	.57***
11. I often take the opportunity to meet people I find attractive.	.02	.54***
15. The possibility of social advancement moves me to action, even if this involves not playing fair.	.25***	.33**
17. I generally give preference to those activities that imply an immediate gain.	.03	.52***
19. I often have trouble resisting the temptation of doing forbidden things.	–.09	.38***
21. I like to compete and do everything I can to win.	–.11**	.36***
29. I sometimes do things for quick gains.	–.01	.56***
33. I would take risks to get rewarded.	–.08	.52***
35. I like to put competitive ingredients in all of my activities.	–.04	.49***
37. I would like to be a socially powerful person.	.13*	.45***
39. I like displaying my physical abilities, even though this may involve danger.	–.20***	.60***
41. When I get something I really want, I feel excited and full of energy.	.04	.31***
43. I like exciting and new activities.	–.23***	.44***
2. I often refrain from doing something because I am afraid of it being illegal.	.52***	–.03
6. I am often afraid of new or unexpected situations.	.64***	–.05
8. It is difficult for me to telephone someone I do not know.	.43***	–.06
10. I often renounce my rights when I know I can avoid a quarrel with a person or an organization.	.48***	.09*
12. I am troubled by punishments.	.44***	.15**
16. I am easily discouraged in difficult situations.	.62***	.06
18. I am a shy person.	.59***	–.20***
20. I avoid demonstrating my skill for fear of being embarrassed.	.64***	.01
22. When I am with a group, I have difficulties selecting a good topic to talk about.	.53***	–.01
24. I is often difficult for me to fall asleep when I think about things I have done or must do.	.46***	.01
26. I would be bothered if I had to return to a store when I noticed I was given the wrong change.	.38***	–.03
28. Whenever I can, I avoid going to unknown places.	.46***	.10*
30. I am often worried by things that I said or did.	.59***	.12**
32. I generally try to avoid speaking in public.	.52***	–.12**
34. I think that I could do more things if it was not for my insecurity or fear.	.71***	–.04
36. Comparing myself to people I know, I am afraid of many things.	.73***	–.07
38. I often find myself worrying about things to the extent that performance in intellectual abilities is impaired.	.46***	.24***
40. I often refrain from doing something I like in order not to be rejected or disapproved of by others.	.49***	.13*
42. Generally, I pay more attention to threats than to pleasant events.	.37***	.22***
44. I often refrain from doing something because of my fear of being embarrassed.	.73***	.01

*Note.* Loading on expected factor is printed in bold. The two-factor solution of the modified SPSRQ included 36 items after deleting item 4, 7, 13, 14, 23, 25, 27, and 31 from the initial 44 items. Additionally, 7 correlated errors were added. SPSRQ, Sensitivity to Punishment and Sensitivity to Reward Questionnaire *p < .05; **p < .01; ***p < .001.

### Reliability

**BIS/BAS Scales.** The internal consistencies of the full BIS- and BAS-scales were .75 and .72 respectively. After the removal of items 4, 5, 10, 18, 22, and 23, the internal consistency of the modified BIS- and BAS-scales slightly decreased to .74 and .70 respectively.

**SPSRQ.** The internal consistencies of the full SP- and SR-scales were .88 and .81 respectively. After the removal of the problematic items 4, 7, 13, 14, 23, 25, 27 and 31, the internal consistency increased to .89 for the modified SP-scale and remained .81 for the modified SR-scale.

### Convergent Validity

BIS and SP of the modified BIS/BAS Scales and SPSRQ were significantly positively related to each other as well as to Harm Avoidance. BAS and SR of the modified BIS/BAS Scales and SPSRQ were significantly positively related to each other as well as to Novelty Seeking. Additionally, the BIS scale of the modified BIS/BAS was significantly positively associated with Reward Dependence and Persistence. No significant correlation was found between BIS and BAS of the modified BIS/BAS Scales, and between SP and SR of the modified SPSRQ. Table 4 presents all correlations.

**Table 4:** Descriptive statistics (means and standard deviations) and Pearson correlations between the scales of the modified BIS/BAS Scales (reduced to 14 items), the modified SPSRQ (reduced to 36 items), and the TCI-SF.

	BIS	BAS	SP	SR	HA	NS	RD	P

BIS	17.70 (3.37)							
BAS	–.04	23.61 (3.43)						
SP	.56***	–.12**	55.34 (12.54)					
SR	–.05	.49***	–.01	46.95 (9.05)				
HA	.57***	–.21***	.68***	–.17**	7.09 (3.96)			
NS	–.22***	.30***	–.36***	.25***	–.40***	7.34 (3.13)		
RD	.23***	–.03	–.01	–.13*	.07	.00	9.10 (2.69)	
P	.13*	.02	–.06	.04	.00	–.24***	.10	7.49 (3.09)

*Note.* BIS = Behavioural Inhibition System, BAS = Behavioural Activation System, SP = Sensitivity to Punishment, SR = Sensitivity to Reward, HA = Harm Avoidance, NS = Novelty Seeking, RD = Reward Dependence, P = Persistence; Means (and standard deviations) are on the diagonal. *p < .05, **p < .01, ***p < .001.

## Discussion

The BIS/BAS Scales (Carver & White, 1994) and the SPSRQ (Torrubia et al., 2001) are considered to be the two most prominent instruments to operationalize Gray’s RST-concepts (Cogswell et al., 2006). However, few studies have examined their psychometric properties among adolescents. Therefore, we examined the internal structure, reliability and convergent validity of the BIS/BAS Scales and the SPSRQ adapted for adolescents in a large adolescent community sample.

In the current sample, we found no conclusive evidence for the two-factor structure of the initial full item questionnaires. Three of the four fit statistics indicated a reasonable fit for the initial two-factor models of both questionnaires. After removal of problematic items, and the addition of correlated errors in the measurement models, all fit indices indicated a good fit for the BIS/BAS Scales. These results are in line with the theoretical assumptions of the RST (Gray, 1970, 1987; Gray & McNaughton, 2000), and previous findings obtained in Dutch adolescent samples (Yu et al., 2011). In the case of the SPSRQ, three of the four fit indices pointed to a good to very good model fit, while a fourth fit index was slightly below the cut-off score of an adequate model fit. Taking all fit indices into account, it seems reasonable to conclude that acceptable fit has been obtained. Regarding the poorly loading items of both questionnaires, several of them have been found to be problematic in previous research as well (Cogswell et al., 2006; Heubeck, Wilkinson, & Cologon, 1998; Jorm et al., 1998; Torrubia et al., 2001; Yu et al., 2011).

The internal consistencies of the modified scales were acceptable for the BIS/BAS Scales and good for the SPSRQ. Regarding construct validity of the modified scales, the relations were mainly in line with the expectations, with significant positive associations between the BIS and SP, and between BAS and SR. Moreover, BIS and SP were also positively associated with Harm Avoidance, and BAS and SR were positively associated with Novelty Seeking. Except for a significant negative correlation between BAS and SP, no other significant correlations were found between measures of the two different theoretical constructs. The non-significant correlations between scales assessing BAS functioning and scales assessing BIS functioning are in line with the original RST suggesting that BIS and BAS are orthogonal and unrelated systems. The significant correlation between BAS and SP supports the Joint Subsystem Hypothesis (Corr, 2001, 2004) suggesting that behavioural and emotional outcomes may be the result of the combined BIS and BAS sensitivities (Corr, 2001, 2004). Unexpectedly, we found that the BIS-scale was significantly and positively related to Persistence. Since the ‘drive’ component of the BAS-scale is theoretically related to Persistence, a positive correlation was expected between Persistence and the BAS-scale instead of the BIS-scale (Mardaga & Hansenne, 2007). It would be relevant to investigate whether this finding is age-specific, culture specific or instrument-specific. It is also possible that Persistence is not uniquely associated with the BAS or with the BIS, but depends on the combination of both.

This study has several strengths, such as the use of a large community sample of adolescents and the inclusion of both the BIS/BAS Scales and the SPSRQ. However, several weaknesses have to be noted as well. The distribution of educational level in the study sample differed from the Flemish adolescent population (i.e., 40% general education; 31% technical education and 25% vocational education, “Vlaams Onderwijs in Cijfers,” 2014–2015) as indicated by the high and low number of adolescents in general education and vocational education, respectively. Future research should replicate the current study in a representative sample. It is further unsure to what extent people are able to report on BIS and BAS sensitivities themselves (e.g., Smillie et al., 2006). Further research is needed to clarify this issue and may show the necessity to develop behavioural measures of BIS and BAS. This is important given the various domains in which BIS and BAS sensitivity plays a role in adolescence, such as risk taking behaviour (e.g., Romer & Hennessy, 2007) and eating disorders (e.g., Matton et al., 2013, 2015). Furthermore, both the BIS/BAS Scales and the SPSRQ are based on the original RST. Future research should incorporate the adaptations of the revised RST to achieve a more specific distinction between motivated behaviours (Heym et al., 2008; Smillie et al., 2006).

In conclusion, the present results indicate some psychometric problems in the full item sets of BIS/BAS Scales and SPSRQ. Moreover, the results show that the use of a two-factor model to analyze data gathered with the modified BIS/BAS Scales (reduced to 14 items) or modified SPSRQ (reduced to 36 items) is more appropriate, although there is still room for improvement. Based on the internal consistencies and convergent validity of the 14-item BIS/BAS Scales and the 36-item SPSRQ in the current study, both modified questionnaires are considered valid and reliable indices of BIS and BAS sensitivity in Flemish adolescents. For future research in Flemish adolescents, we strongly recommend to use the questionnaires without the malfunctioning items.

## Additional Files

The additional files for this article can be found as follows:

- **Additional file 1: Appendix.** Dutch version of the Sensitivity to Punishment and Sensitivity to Reward Questionnaire (Verbeken, 2009). pb-56-4-298-s1.pdf
  DOI: http://dx.doi.org/10.5334/pb.298.s1
