# Supplementary material for: Psychometric Properties of the BIS/BAS Scales and the SPSRQ in Flemish Adolescents
Source: Psychol Belg. 2016 Dec 20;56(4):406–20. doi: 10.5334/pb.298 (PMC5853848; doi:10.5334/pb.298)
Supplement: Supplementary file 1 [file pb-56-4-298-s1.pdf]

Appendix A

Dutch version of the Sensitivity to Punishment and Sensitivity to Reward Questionnaire (Verbeken, 2009)

1. Als ik weet dat ik een beloning kan krijgen voor een taak, ben ik meer gemotiveerd om eraan te beginnen
2. Ik hou mij vaak in om dingen te doen omdat ik bang ben dat het niet mag
3. Ik sta graag in het centrum van de belangstelling
4. *Ik kies ervoor iets niet te vragen als het niet zeker is dat ik het zal krijgen*
5. Ik spendeer veel tijd aan mijn imago
6. Ik ben bang van nieuwe en onverwachte situaties
7. *Ik heb mensen nodig die me voortdurend laten zien dat ze me graag hebben*
8. Ik vind het moeilijk om naar iemand te bellen die ik niet ken
9. In groep probeer ik over te komen als de slimste of de grappigste
10. Ik geef vaak mijn rechten op om conflicten met anderen te vermijden
11. Ik probeer zoveel mogelijk om te gaan met mensen die ik aantrekkelijk vind
12. Ik ben van streek als ik gestraft word
13. *Ik doe vaak dingen om de goedkeuring van anderen te krijgen*
14. *Als ik niet voorbereid ben op een taak, denk ik steeds dat het niet goed zal aflopen*
15. Ik doe er alles aan opdat mensen me leuk zouden vinden, ook al ben ik dan niet altijd oprecht
16. Ik verlies gemakkelijk de moed in moeilijke situaties
17. Ik doe het liefst dingen waarvoor ik onmiddellijk een beloning kan krijgen

18. Ik ben een verlegen persoon
19. Ik kan moeilijk aan de verleiding weerstaan om verboden dingen te doen
20. Ik toon niet vaak waar ik goed in ben omdat ik me zou schamen
21. Ik hou van competitie en doe er alles aan om te winnen
22. In groep vind ik het moeilijk om een goed gespreksonderwerp te vinden
23. *Ik kan gemakkelijk smaken en geuren in verband brengen met aangename gebeurtenissen*
24. Ik geraak moeilijk in slaap als ik denk aan dingen die ik gedaan heb of die ik nog moet doen
25. *Er zijn veel dingen en gevoelens die mij herinneren aan plezierige gebeurtenissen*
26. Ik zou mij vervelend voelen als ik terug moet naar een winkel omdat ze me te weinig geld hebben terug gegeven
27. *Ik kan moeilijk stoppen met een leuke activiteit*
28. Ik probeer zo weinig mogelijk naar ongekende plaatsen te gaan
29. Ik doe soms dingen voor het krijgen van snelle winst of beloning
30. Ik maak mij vaak zorgen over dingen die ik zei
31. *Ik vind het moeilijk om mij te concentreren op een taak als er iets leuker is dat ik kan gaan doen*
32. Ik vermijd zoveel mogelijk om in groep te spreken
33. Ik zou risico’s nemen om een beloning te krijgen
34. Ik denk dat ik meer dingen zou kunnen als ik niet zo onzeker of angstig was
35. Ik hou ervan om competitie te maken van alles wat ik doe
36. In vergelijking met anderen ben ik bang van vele dingen

37. Ik zou graag een sociaal machtig persoon zijn
38. Ik maak mij vaak zoveel zorgen dat ik hierdoor niet meer zo goed kan nadenken en niet zo goede punten haal op school
39. Ik toon graag wat ik kan, ook al kan dit gevaar inhouden
40. Ik doe vaak dingen niet omdat ik bang ben voor verwerping of afkeuring door anderen
41. Als ik iets krijg wat ik heel graag wil, voel ik mij opgewonden en vol energie
42. Ik heb algemeen meer aandacht voor bedreigingen dan voor leuke dingen
43. Ik verlang sterk naar spannend en nieuwe activiteiten
44. Ik doe vaak dingen niet omdat ik anders beschaamd zou zijn

Note. Every item is rated on a 5-point Likert scale: “Nooit”, “Zelden”, “Soms”, “Vaak”, “Altijd”

Problematic items are printed in italic.

SP-scale: items 2, 4, 6, 8, 10, 12, 14, 16, 18, 20, 22, 24, 26, 28, 30, 32, 34, 36, 38, 40, 42, 44

SR-scale: items 1, 3, 5, 7, 9, 11, 13, 15, 17, 19, 21, 23, 25, 27, 29, 31, 33, 35, 37, 39, 41, 43
